# Supplementary material for: Challenges for Cryptosporidium Population Studies
Source: Genes (Basel). 2021 Jun 10;12(6):894. doi: 10.3390/genes12060894 (PMC8229070; doi:10.3390/genes12060894)
Supplement: Supplementary file 1 [file genes-12-00894-s001.zip › genes-1226889-suppl/Supplemental Methods.pdf]

SUPPLEMENTAL METHODS

Challenges for *Cryptosporidium* Population Studies

Rodrigo P. Baptista [1,2,*], Garrett W. Cooper [3] and Jessica C. Kissinger [1,2,3]

Methods used to generate Figures 1A and 1B.

1. Phylogenetic analysis

Nucleotide sequences retrieved from GenBank (Supplemental Table S1), were aligned using MAFFT v7 [1], and had poorly aligned regions removed with trimAl v1.4.1 [2]. The final alignment was then submitted to Modeltest-NG [3] for best substitution model selection and the maximum likelihood tree was reconstructed using PhyML v3.3 [4] with 1,000 bootstrap replications.

2. Admixture analysis

Illumina sequence reads from 31 different isolates of *C. hominis* from different *gp60* subtypes (Supplemental Table S2) were aligned against the *C. parvum* IOWA-ATCC reference genome sequence using BWA-MEM v0.7.17 [5], the bam files were parsed to select uniquely mapped reads and to mark duplicates and remove redundancy using PICARD v2.21.6 [6] and then submitted to a variant call analysis using GATK v3.8 Haplotypecaller [7]. These results were then filtered by mapping quality > 40 and depth coverage >10. Because mixed infections exist and to avoid their impact in the analysis, we restricted the analysis to biallelic sites. The individual VCF files were combined into one GVCF file using the GATK tools CombineGVCF and GenotypeGVCF. To ensure all downstream analysis contained only single-nucleotide polymorphisms, all indels were removed from the analysis using VCFtools v0.1.15 [8], and then converted to the binary BED format using PLINK v1.9 [9]. The software tool ADMXITURE v1.3.0 [10] was then used to estimate the maximum likelihood of individual ancestries. The cross-validation error flag (–cv) was used to calculate the statistical likelihood of each K-value.

REFERENCES

1. Katoh, K.; Standley, D.M. MAFFT multiple sequence alignment software version 7: improvements in performance and usability. *Mol Biol Evol* **2013**, *30*, 772–780, doi:10.1093/molbev/mst010.
2. Capella-Gutierrez, S.; Silla-Martinez, J.M.; Gabaldon, T. trimAl: a tool for automated alignment trimming in large-scale phylogenetic analyses. *Bioinformatics (Oxford, England)* **2009**, *25*, 1972–1973, doi:10.1093/bioinformatics/btp348.
3. Darriba, D.; Posada, D.; Kozlov, A.M.; Stamatakis, A.; Morel, B.; Flouri, T. ModelTest-NG: A New and Scalable Tool for the Selection of DNA and Protein Evolutionary Models. *Mol Biol Evol* **2020**, *37*, 291–294, doi:10.1093/molbev/msz189.

4. Guindon, S.; Dufayard, J.F.; Lefort, V.; Anisimova, M.; Hordijk, W.; Gascuel, O. New algorithms and methods to estimate maximum-likelihood phylogenies: assessing the performance of PhyML 3.0. *Syst Biol* **2010**, *59*, 307–321, doi:10.1093/sysbio/syq010.
5. Li, H.; Durbin, R. Fast and accurate short read alignment with Burrows-Wheeler transform. *Bioinformatics (Oxford, England)* **2009**, *25*, 1754–1760, doi:10.1093/bioinformatics/btp324.
6. Broad_Institute. Picard Tools. Available online: http://broadinstitute.github.io/picard/]. (accessed on 6 March 2021).
7. McKenna, A.; Hanna, M.; Banks, E.; Sivachenko, A.; Cibulskis, K.; Kernytsky, A.; Garimella, K.; Altshuler, D.; Gabriel, S.; Daly, M.; et al. The Genome Analysis Toolkit: a MapReduce framework for analyzing next-generation DNA sequencing data. *Genome Res* **2010**, *20*, 1297–1303, doi:10.1101/gr.107524.110.
8. Danecek, P.; Auton, A.; Abecasis, G.; Albers, C.A.; Banks, E.; DePristo, M.A.; Handsaker, R.E.; Lunter, G.; Marth, G.T.; Sherry, S.T.; et al. The variant call format and VCFtools. *Bioinformatics (Oxford, England)* **2011**, *27*, 2156–2158, doi:10.1093/bioinformatics/btr330.
9. Purcell, S.; Neale, B.; Todd-Brown, K.; Thomas, L.; Ferreira, M.A.; Bender, D.; Maller, J.; Sklar, P.; de Bakker, P.I.; Daly, M.J.; et al. PLINK: a tool set for whole-genome association and population-based linkage analyses. *American journal of human genetics* **2007**, *81*, 559–575, doi:10.1086/519795.
10. Alexander, D.H.; Lange, K. Enhancements to the ADMIXTURE algorithm for individual ancestry estimation. *BMC Bioinformatics* **2011**, *12*, 246, doi:10.1186/1471-2105-12-246.
